# Recent Application of Artificial Intelligence in Non-Gynecological Cancer Cytopathology: A Systematic Review

**DOI:** 10.3390/cancers14143529

**Published:** 2022-07-20

**Authors:** Nishant Thakur, Mohammad Rizwan Alam, Jamshid Abdul-Ghafar, Yosep Chong

**Affiliations:** Department of Hospital Pathology, College of Medicine, The Catholic University of Korea, Seoul 06591, Korea; nishantbiotech2014@gmail.com (N.T.); rizwan@catholic.ac.kr (M.R.A.); jamshid@catholic.ac.kr (J.A.-G.)

**Keywords:** artificial intelligence, cytopathology, cancer, deep learning, systematic review

## Abstract

**Simple Summary:**

Artificial intelligence (AI) has attracted significant interest in the healthcare sector due to its promising results. Cytological examination is a critical step in the initial diagnosis of cancer. Here, we conducted a systematic review with quantitative analysis to understand the current status of AI applications in non-gynecological (non-GYN) cancer cytology. In our analysis, we found that most of the studies focused on classification and segmentation tasks. Overall, AI showed promising results for non-GYN cancer cytopathology analysis. However, the lack of well-annotated, large-scale datasets with Z-stacking and external cross-validation was the major limitation across all studies.

**Abstract:**

State-of-the-art artificial intelligence (AI) has recently gained considerable interest in the healthcare sector and has provided solutions to problems through automated diagnosis. Cytological examination is a crucial step in the initial diagnosis of cancer, although it shows limited diagnostic efficacy. Recently, AI applications in the processing of cytopathological images have shown promising results despite the elementary level of the technology. Here, we performed a systematic review with a quantitative analysis of recent AI applications in non-gynecological (non-GYN) cancer cytology to understand the current technical status. We searched the major online databases, including MEDLINE, Cochrane Library, and EMBASE, for relevant English articles published from January 2010 to January 2021. The searched query terms were: “artificial intelligence”, “image processing”, “deep learning”, “cytopathology”, and “fine-needle aspiration cytology.” Out of 17,000 studies, only 26 studies (26 models) were included in the full-text review, whereas 13 studies were included for quantitative analysis. There were eight classes of AI models treated of according to target organs: thyroid (*n* = 11, 39%), urinary bladder (*n* = 6, 21%), lung (*n* = 4, 14%), breast (*n* = 2, 7%), pleural effusion (*n* = 2, 7%), ovary (*n* = 1, 4%), pancreas (*n* = 1, 4%), and prostate (*n* = 1, 4). Most of the studies focused on classification and segmentation tasks. Although most of the studies showed impressive results, the sizes of the training and validation datasets were limited. Overall, AI is also promising for non-GYN cancer cytopathology analysis, such as pathology or gynecological cytology. However, the lack of well-annotated, large-scale datasets with Z-stacking and external cross-validation was the major limitation found across all studies. Future studies with larger datasets with high-quality annotations and external validation are required.

## 1. Introduction

Recently, artificial intelligence (AI) has attracted considerable interest in the healthcare sector by providing solutions to problems through automated diagnosis [1,2,3,4,5,6,7,8]. AI approaches, including machine learning (ML) and deep learning (DL), have been used in radiological diagnosis [9], bioinformatics [10], genome sequencing [11], drug development [12], and histopathological image analysis [5,13,14,15]. In histopathological diagnosis, it has been revealed that the accuracy of AI models is similar to or higher than that of expert pathologists in terms of tumor detection and classification [16]. In the CAMELYON Grand Challenge 2016, Liu et al. used a convolutional neural network (CNN) model and detected tumor metastasis with a higher sensitivity than expert pathologists (92.4% vs. 73.2%) [17]. However, the application of AI models in pathology has mainly focused on histopathology rather than cytopathology so far.

Cytological examination is an old but still very popular technique for initial screening diagnosis of cancer, although it shows limited diagnostic efficacy. The examination is rapid, minimally invasive, easy to use, cheap, and easily repeatable [18,19], making it the most suitable test method for the screening of cancer. However, it is less accurate, as well as labor-intensive and time-consuming, compared to histopathological examination. Sampling inadequacy (low cellularity or bloody smear), cellular degeneration and artifacts, inter-examiner variation in sampling and preparation procedures, and inter-observer variation in interpretation are the major reasons for the suboptimal sensitivity and specificity [18,19]. In addition, contrary to histopathology, cellular materials that are critical for the diagnosis are often dispersed in the smearing field, requiring more time and labor to examine whole slides [18]. Therefore, there is a need for the application of new techniques, such as AI image analysis, to increase the accuracy of the test and reduce labor and time costs.

The application of AI models has been exploited in the field of cytology, firstly, in the examination of gynecological (GYN) samples, with promising results, and is now gradually being expanded to non-GYN samples [20]. A recent multicenter study conducted on 2145 referral women reported a higher specificity of the supervised DL model compared to experienced cytologists [21]. Recently, its application has expanded to non-GYN samples. In 2020, Range et al. developed an ML-based model using 908 whole-slide images (WSIs) of fine-needle aspiration cytology (FNAC) of the thyroid. Their model could predict the risk of malignancy in the thyroid of patients with comparable sensitivity, specificity, and areas under the curve (AUCs) to those of cytopathologists [22]. In 2017, Japanese researchers developed a DL-based model in liquid-based cytology (LBC) of respiratory tract fluids to differentiate three subtypes of lung cancer, including squamous cell carcinoma (SqCC), adenocarcinoma (AdC), and small cell carcinoma (SCLC), directly from the liquid-based cytology (LBC). The accuracy of the initial model by this group was 71.1%, and the consequent models in the following years showed even better performances [23].

However, each study seemed to have several limitations, such as lack of Z-stacking; limited numbers of training, validation, and test datasets for generalization; lack of external validation datasets; and flawed study design and inappropriate levels of evidence—this despite the number of publications increasing every year. Moreover, no relevant review has been published to date. Thus, we designed a systematic review of state-of-the-art AI applications in non-GYN cancer cytology to understand the current technical status and performed a qualitative analysis to set out the basic requirements for future study designs.

## 2. Materials and Methods

The present systematic review and meta-analysis follows the guidelines set out in the PRISMA (Preferred Reporting Items for Systematic Reviews and Meta-Analysis) statement [19]. The guidelines for this systematic review were registered in the PROSPERO database (CRD42021281330).

### 2.1. Literature Search

This study was approved by the Institutional Review Board of the Catholic University of Korea, College of Medicine (UC21ZISI0053). We searched the three major electronic databases, MEDLINE, Cochrane, and EMBASE, for articles published between January 2010 and January 2021 written in the English language. The query terms used in the search were: “deep learning”, “cytopathology”, “non-gynecological cancers”, “image processing”, “cell biology”, “lung cancer”, “deep learning”, “image processing”, “computer-assisted”, “machine learning”, “lung cytopathological deep learning”, “urine cytology deep learning”, and “thyroid cytological machine learning”. Further, we also obtained articles by cross-referencing the keywords. Subsequently, we removed the articles without full texts available and then retrieved and managed the remaining articles with EndNote X20 (Bld 10136, Thomson Reuters, New York, NY, USA).

### 2.2. Study Selection, Reviewing, and Data Retrieval

**Figure 1:**
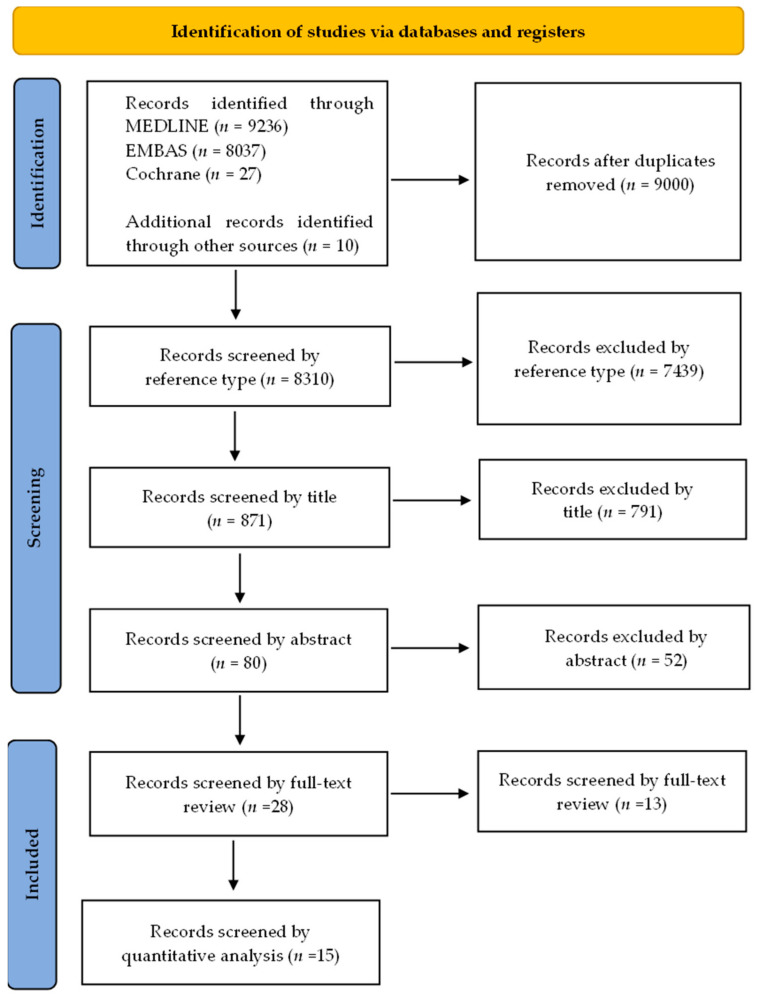
PRISMA flow chart showing the study selection process.

Figure 1 explains the criteria used for selecting and reviewing the articles. After the initial search, duplicate studies were excluded from the results after the preliminary search. Next, the titles and abstracts of the studies were screened (N.T.). If any discordance occurred, a second reviewer stepped in and a consensus was reached (Y.C.). Case reports, conference proceedings, letters, reviews, and posters were removed. Only original studies with full texts on AI in non-gynecological cancer cytological image analysis were included. In addition, the references cited in the individual study were manually searched and reviewed to identify any supplementary studies.

## 3. Results

### 3.1. Study Selection and Characteristics

The article selection process for this systematic review is shown in Figure 1. In the present study, 17,310 articles were identified in the primary search, including 3815 from MEDLINE (PubMed), 4953 from EMBASE, 202 from Cochrane library, and 10 from cross-referencing. After excluding 9000 duplicate articles, a total of 8310 articles remained. After excluding 7439 articles based on reference types, 871 articles were screened. A total of 791 articles were removed based on the title, and 80 articles were screened by abstract. Next, 52 articles were removed, and 28 studies were eligible for full-text review analysis. After removing 13 articles, only 15 articles fulfilled the criteria for quantitative analysis.

### 3.2. Applications of AI in Non-Gynecological Cancer Cytology Image Analysis

**Figure 2:**
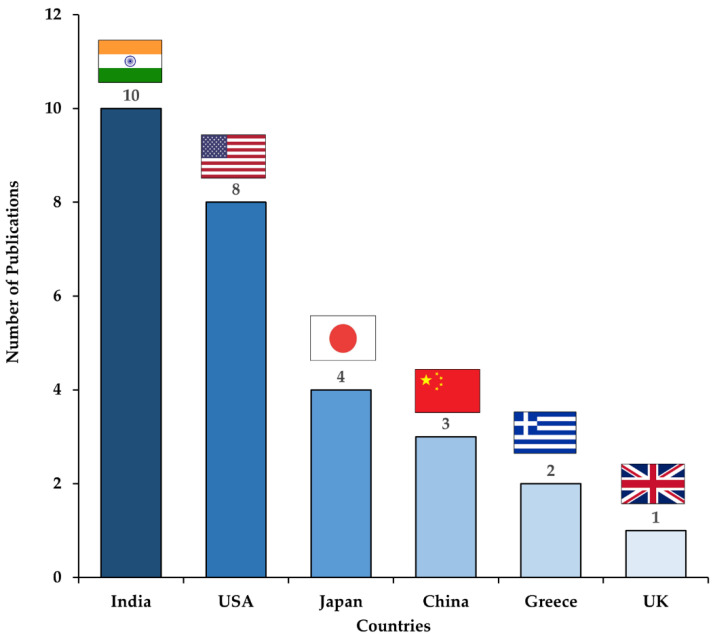
Number of publications of AI models on cytopathological image analysis by country.

**Figure 3:**
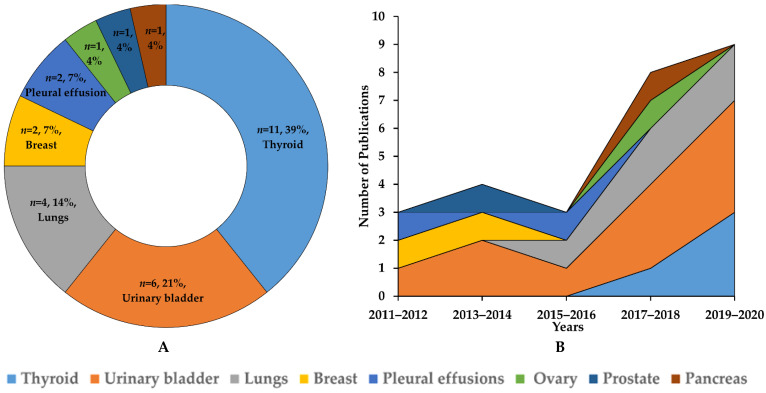
Classification of artificial intelligence models for cytopathological image analysis according to target organ from 2010 to 2020 (**A**) along with yearly trends (**B**).

The characteristics of the AI models in non-GYN cancer cytology are summarized in Table 1. All 26 studies were published from March 2010 to March 2021 and conducted universally, including nine models in India, seven models in the USA, four models in Japan, three models each in China, two models in Greece, and one model in the UK, as shown in Figure 2 [22,23,24,25,26,27,28,29,30,31,32,33,34,35,36,37,38,39,40,41,42,43,44,45,46,47,48,49]. Further, we classified the algorithmic features of the models with respect to eight target organs: thyroid (*n* = 11, 39%); urinary bladder (*n* = 6, 21%); lung (*n* = 4, 14%); breast (*n* = 2, 7%); pleural effusion (*n* = 2, 7%); ovary (*n* = 1, 4%); pancreas (*n* = 1, 4%); and prostate (*n* = 1, 4%) (Figure 3A). The number of published articles is increasing every year, especially those on the thyroid, urinary bladder, and lung (Figure 3B). This section is divided by subheadings. It should provide a concise and precise description of the experimental results, their interpretation, as well as the experimental conclusions that can be drawn.

#### 3.2.1. Classification for Thyroid FNAC

**Figure 4:**
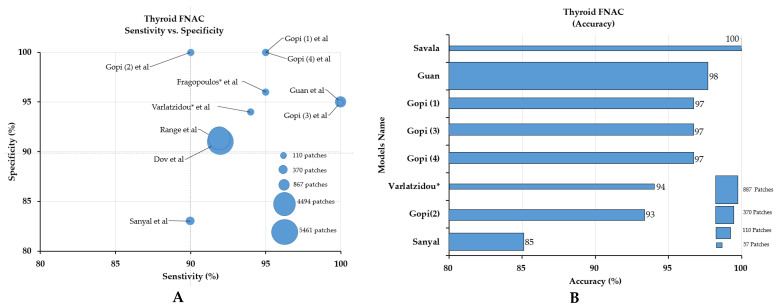
Comparison of sensitivity, specificity (**A**), and accuracy (**B**) of AI classification models in thyroid fine-needle aspiration cytology. The models by Gopinath (3) [36], and Guan et al. [40] showed higher sensitivity; the model by Gopinath (1) [34], Gopinath (2) [35], and Gopinath (4) [49] et al. showed higher specificity; and the model by Savala et al. [37] showed higher accuracy. * Indicates that the dataset was in nuclei form instead of patches.

In the classification category of the thyroid, nine studies were included and classified as papillary thyroid carcinoma (PTCA), benign thyroid nodules (BTNs), follicular adenoma (FA), and follicular carcinoma (FC). The size of the datasets ranged from 279 to 908 WSIs [31,32,33,34,35,36,37,38,45]. Most of the studies did not provide detailed information on the participating pathologists. By comparing the sensitivity, specificity, and accuracy of the models, the models by Gopinath (3) [36] and Guan et al. [40] showed the best sensitivity, the first, second, and fourth models by Gopinath et al. [34,35,49] showed the best specificity (Figure 4A), and the model by Savala et al. showed the best accuracy (100%, Figure 4B) [37].

#### 3.2.2. Classification for Urinary Tract Cytology

Six studies were included in the cytology classification of urine, and the dataset used ranged from 49 image patches to 2405 WSIs [42,43,44,45,46,47]. The AI models were trained to classify the urine samples of patients into three to four histological types, such as benign, low-grade, and high-grade urothelial carcinomas, or to classify the cell clusters into specific cell types, such as benign, atypical, and malignant urothelial cells, squamous cells, crystals, erythrocytes, leukocytes, blurry images, debris, degenerated cells, and inflammatory cells (Table 1). Most studies in this group used the DL model and focused on classification tasks. None of the studies used an external validation dataset to check the robustness of the models. Although the dataset was limited in each study, the performance of the models was very high, with AUCs reaching up to 0.989, F1 scores reaching up to 0.900, sensitivity scores up to 79.5%, specificity scores up to 84.5%, and accuracy scores up to 99.1% (Table 1). Direct comparison of the models was impossible because each study had an individual focus and used different performance metrics.

#### 3.2.3. Classification for Lung FNAC or Bronchoscopic (Respiratory Tract) Aspirates

**Figure 5:**
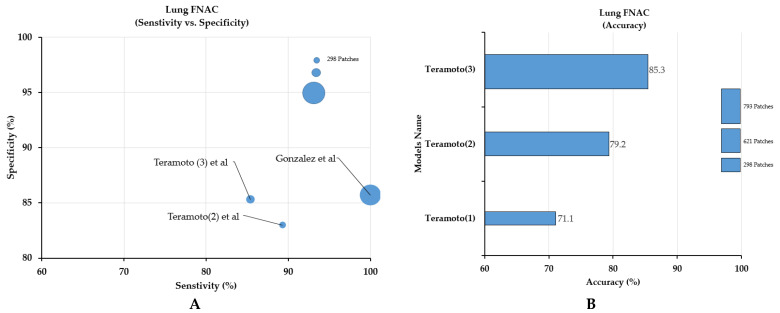
Comparison of sensitivity, specificity (**A**), and accuracy of models (**B**) with respect to lung cytology samples. The model by Gonzalez et al. [28] showed the best sensitivity and specificity, while the models by Teramoto et al. [23] showed the best accuracy.

In the lung cancer category, four studies using DL-based models were included. To construct the model for the classification of lung cancers into two to five histological subtypes, such as benign, ADC, SqCC, SCLC, and large cell neuroendocrine carcinoma (LCNEC), 117 to 298 WSIs (621-46,4378 patch images) were used [23,26,27]. In all studies, sampling of the specimen was performed using either computed tomography (CT)-guided FNAC or bronchoscopy. None of the studies disclosed information regarding the annotation process and involvement of the pathologists during sample selection. Although the dataset used in each study was different, the model by Gonzalez et al. [28] performed the best in terms of sensitivity and specificity, while the third model by Teramoto et al. [23] performed the best in terms of accuracy (Figure 5).

#### 3.2.4. Classification for Breast FNAC

Only two models were identified in the FNAC classification category for the breast. In 2011, Dey et al. developed an artificial neural network (ANN) model using 64 image patches and successfully differentiated all benign and invasive lobular cancer (ILC) cases and six out of seven invasive ductal carcinoma (IDC) cases [24]. In 2013, Subbaiah et al. developed an ANN model using 112 image patches and classified fibroadenoma and IDC based on nuclear morphometric and densitometric features with 100% sensitivity and specificity [25].

#### 3.2.5. Classification for Pleural Fluids

In 2011, Barwad et al., constructed an ANN model using 114 image patches of pleural fluids for the classification of benign and metastatic cancer based on cytological features, image morphometric data, densitometric data, and chromatin textural data, and successfully differentiated cancer cases with an AUC of 1.00 [28]. In 2015, Tosun et al. used 1080 nuclei from 34 pleural fluid cases to develop a DL model to differentiate mesothelioma from normal mesothelial cells and achieved 100% sensitivity and specificity for segmentation and classification [28].

#### 3.2.6. Classification for Ovary FNAC

**Figure 6:**
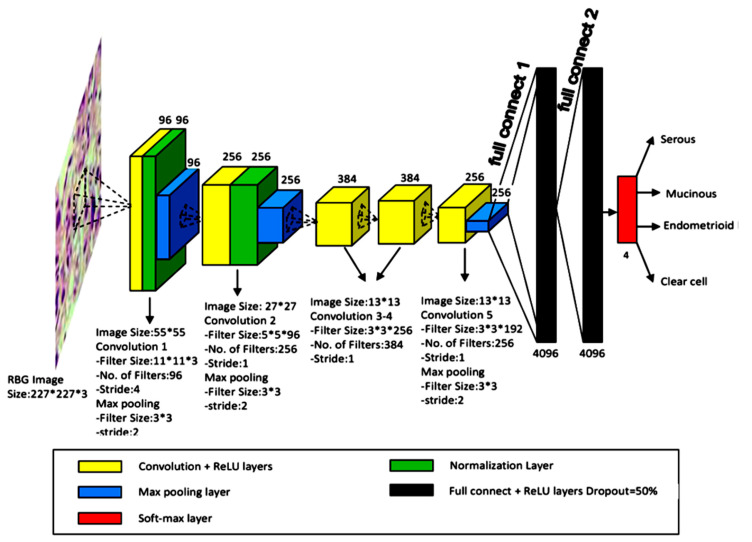
Example of cytological classification for ovarian cancer. The architecture and illustration of CNN for ovarian cancer image classification. (Source: Figure 4 in Wu et al. [29]. Reprinted with permission from the authors. Copyright (2019), Bioscience Reports, Portland Press [29].).

In 2018, Wu et al., trained an AlexNet model on 85 WSIs of FNAC of the ovary and augmented these images into 20,328 patch images for the classification of ovarian cancer into histological subtypes, such as serous carcinoma, mucinous carcinoma, endometrioid carcinoma, and clear cell carcinoma, with an accuracy of 78.20% [29]. An overview of the study design is shown in Figure 6.

#### 3.2.7. FNAC Classification for the Pancreas

Only one model by Boroujeni et al. used a k-means clustering algorithm for the segmentation of cell clusters into individual spots. Then, a multilayer perceptron neural network (MNN) was trained on 277 images of FNAC of the pancreas for the classification of benign, atypical, and malignant cases. The model achieved 100% accuracy for benign and malignant cases but only 77% accuracy for atypical cases. Moreover, they found that their model predicted better survival outcomes in benign cases [30].

#### 3.2.8. FNAC Classification for the Prostate

For FNAC classification of the prostate, Nguyen et al., developed a support vector machine (SVM) with a radial basis function (RBF) kernel on a 17 WSI dataset (6 for training and 11 for the test) for the segmentation and classification of benign and cancer cases based on cytological features and texture features with 78% sensitivity [33]

## 4. Discussion

In this systematic review, we found that AI-based models showed impressive accuracy in segmentation and classification tasks in non-GYN cancer cytopathology. However, there are still several challenges that need to be addressed for the implementation of AI in cytological diagnosis, the enumeration of which might be helpful to AI computer scientists who are planning to design a study on this topic.

### 4.1. Challenges in Cytological Diagnosis

**Figure 7:**
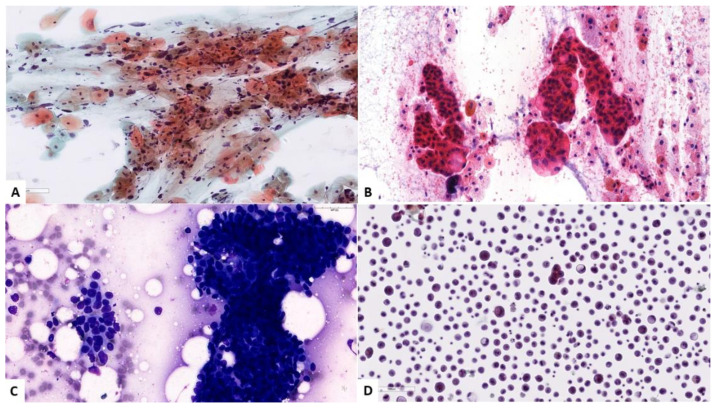
Examples of cytological images. (**A**) Conventional smear from uterine cervix of a 30-year-old female showing high-grade squamous intraepithelial lesion (HSIL) (Pap stain, ×20). (**B**) Conventional smear from a salivary gland mass of a 30-year-old female patient, showing intraductal carcinoma (H&E stain, ×20). (**C**) High-power view of a conventional smear from a 64-year-old male patient with a submandibular gland nodule, showing adenoid cystic carcinoma (Diff-Quik stain, ×40). (**D**) Ascitic fluid liquid-based preparation from a 79-year-old female patient with a history of colon carcinoma showing metastatic colon carcinoma (Pap stain, ×20).

Cytology/cytopathology is the area of pathology that seeks to diagnose disease through the examination of individual or clustered cell characteristics and the histological features of the nuclei and cytoplasm of cells (Figure 7). It was first introduced in the 18th century and rapidly progressed through the invention of the Pap smear or Pap test by Dr. George Papanicolaou in the mid-20th century [19,50]. Thereafter, it gained much attention from various scientific communities and underwent various modifications to be transformed into a standardized protocol. Cytology can be divided into two groups according to the mode of sampling: exfoliative and aspiration. Exfoliative cytology includes bronchial washing, brushing, sputum, bronchoalveolar lavage (BAL), urine and bladder washing, and body cavity fluids, such as pleural, peritoneal, and pericardial fluids [19,50]. Aspiration cytology, also called FNAC/biopsy, can be frequently performed on solid organ tumors, such as tumors of the thyroid, salivary glands, pancreas, breast, and ovary [19,50]. These methods are now widely used for initial cancer screening. In South Korea, over 10 million tests are now being performed nationwide [51]. Since the cytology started from GYN samples, the non-GYN samples accounted for about 24% of all cytological samples [51]. However, the number and proportion of non-GYN samples have been increasing recently [52]. Owing to the widespread use of the Pap smear test, the rate of cervical cancer mortality has significantly decreased in developed countries. There are several advantages to and challenges associated with cytological examinations that need to be discussed in detail.

### 4.2. Challenges of Cytological Exams

Although the advantages of the cytological exam are obvious, such as the rapidity, simplicity, and cost-effectiveness, and make it the most suitable screening method, the limited diagnostic accuracy due to impaired sampling adequacy, the time-consuming and labor-intensive interpretation process, the variability in sampling, the slide-producing process, and inter- and intra-cytopathological interpretation are currently the biggest obstacles to overcome and might be considered good reasons for introducing AI technology in this field.

#### 4.2.1. Sampling Adequacy

Sampling adequacy is an important part of cytological sampling and a common reason for false negative results, often because of the small size of the specimens, the bloody or cystic nature of the targeted lesions (especially in thyroid FNAC), and the differences in sampling techniques and experience between examiners and cytotechnicians. For example, drying artefacts and hypercellular or bloody obscuration have been some of the common problems with the Pap smear procedure which can be caused by smearing technique. To overcome these sampling errors, LBC, often represented by centrifuge smears using membrane filters or monolayer liquid-based cytology (i.e., ThinPrep and SurePath), was developed and is now being widely used in GYN sampling [18,53,54].

#### 4.2.2. Time-Consuming and Labor-Intensive Tasks

Screening and interpreting cytological slides are highly time-consuming and labor-intensive activities because the screening area is much larger than the histological samples and the screening should be carried out under higher magnification at “cell level”. Sometimes, cancer cells can be very scarce, making the screening process like searching for a needle in a haystack. Cancer cells may be missed behind benign cells or obscured by background elements. For these reasons, it may take up to 30–40 min to interpret one slide [50,53]. Therefore, cytology is considered to be more suitable as a screening test.

#### 4.2.3. Intra-Examiner Variation in Sampling Procedures

Sampling procedures may vary from person to person and according to their knowledge. When a target tissue is small, deep-seated, and next to a muscle, the person who is performing the sampling procedure should be well-experienced and able to evaluate whether a sample is from within a lesion or outside a lesion [18,53,55].

#### 4.2.4. Inter- and Intra-Observer Interpretational Variation among Cytologists

As cytological diagnosis is a more qualitative or subjective mode of interpretation than it is a quantitative analysis, there is a possibility of inter- and intra-observer variation among cytologists’ interpretations [50,56]. For example, even though the Paris system (TPS) was established to standardize the cytological interpretation of urine samples, there are still many conflicting opinions about the criteria for some categories. The development of certain diagnostic systems was delayed because it was not an easy to reach agreement among experts [57,58]. For example, in 2012, Reid et al. reported very poor agreement among three pathologists on urine cytology diagnoses, with a coefficient kappa of less than 40% and accumulated grading accuracy of only 77% [59].

### 4.3. Challenges Related to the Application of AI in Cytology

#### 4.3.1. The Larger Size of the Image

A cytological specimen consists of broadly and randomly dispersed cell clusters over whole glass slides [60]. Scanning requires more time due to higher magnification (e.g., ×40) and larger scanning area, resulting in bigger file sizes (2–20 times that of normal histological WSIs) and the need for more computational resources [60,61].

#### 4.3.2. Difficulty in Annotation

Unlike histological images, cell/cluster-level classification or nuclear segmentation is possible in cytological samples. However, this requires cell/cluster level annotation that can be very tedious and time-consuming. What makes this worse is that sometimes it is very hard to set up a ground truth for each cell or cluster. In nuclear segmentation, overlapping cells are hard to differentiate; sometimes pieces of denser tissue and red blood cell contamination hide cancerous cells, or they are obscured by normal cells, making it difficult for cytotechnicians to reach final decisions and requiring the expenditure of more time and resources, with more personnel being needed to annotate larger datasets [60,62].

#### 4.3.3. Limited Z-Stacked Images

Z-axis scanning is obtained through several scans of the same slide captured at various focal planes being assembled into a final combined image. It is always advantageous to access the morphological structures of individual cell clusters through different cell layers for the precise interpretation of cytological cases [63]. However, when scanning is performed with the single Z-plane, the procedure is limited to the achievement of two-dimensional virtual images. Z-axis scanning or three-dimensional (3D) scanning is always necessary to obtain 3D cluster images [63]. A cytological specimen consists of both single cells and 3D cell clusters; most often WSIs are obtained on the single Z-plane, where it is very difficult to concentrate only one depth of field, while with the Z-stacking of images the 3D structure of cells can be obtained and the focus of cells in digital images can be enhanced, which may also improve the performance of algorithms [63]. Several other negative factors, such as overlapping inflammatory cells and blood obscuration, which affect model efficacy, can also be addressed by Z-stacking. However, it is not easy to capture these images and doing so takes more than 30 min, while file sizes may reach up to 11 GB and the storage of such large datasets requires larger servers [63].

#### 4.3.4. Lack of Well-Annotated Larger Datasets

Generally, an AI algorithm relies on a large set of good-quality labelled images. These images are manually annotated by expert cytotechnicians or cytopathologists [62]. The detailed annotation of these images is not only a time-consuming and monotonous task but may also be challenging due to working at low resolutions and with slow networks. In such settings, annotation requires large amounts of computational resources and large numbers of WSIs [62].

#### 4.3.5. Limited Publicly Available Datasets and Grand Challenges

In contrast to cytopathology, the application of AI-based models has been explored more in histopathology because many publicly available sites, such as The Cancer Genome Atlas (TCGA) and GTEx, provide histopathological images and associated annotations [16]. Moreover, many grand challenges, such as the Gland Segmentation in Colon Histology Images (GlaS) challenge, the CAMELYON (Cancer Metastases in Lymph Nodes) grand challenge, etc., provide basic platforms for the researcher along with datasets with which to showcase robust AI models [16,64]. However, almost no grand challenges or large sets of cytology images for non-GYN cancer are publicly available.

#### 4.3.6. Variation in the Annotation of Datasets and Image Quality

For the segmentation of biological structures, the performance of an AI model is dependent on the fidelity of the annotations made by the expert cytotechnician in the learning set. If there is any variation in the annotation dataset, then the model will not perform well and can show errors in the clinical setting during diagnosis [65,66]. Apart from this, AI-based models are significantly dependent upon the quality of images and the datasets used in training should be clean and artefact-free. If images are scanned at low resolution, then the model will be unable to differentiate the detailed information required to evaluate tissues [66].

### 4.4. Evolving Trends of AI Models in Cytology

#### 4.4.1. GYN Cytology

Automating examination of the Papanicolaou smear test has a long history, the primary aim of automatic detection being to reduce the workload of cytopathologists and improve the accuracy of testing. PAPNET was the first commercially available automation-assisted system, designed by Neuromedical Systems, Inc. (NSI), in 1992, for the detection of cervix atypical cells in Pap tests that were missed by manual observation as carried out by cytopathologists [67,68,69]. PAPNET consisted of image-processing and neural network processing units. Firstly, an image-processing algorithm based on morphometry would be run to recognize individual cells and cell clusters through local contrast and grayscale intensity features and then object images would be sent to the neural network for the counting of abnormal cells in the original training set. Thereafter, the 128 atypical color images would be recorded onto digital tape and then sent to the cytopathologist for reviewing of the slides [68,69]. Next, if there were any abnormalities discovered in the slides, then the cytopathologist would perform a further examination under a conventional microscope. After PAPNET, the next automated examination system to appear was the ThinPrep Imaging System, which was FDA-approved in 2004 and promptly embraced by the big laboratories [68,69]. The system was trained on a proprietary algorithm to examine single and clustered cells, which identify the most pertinent fields of view (FOVs) and then data was stored in the computer. Thereafter, a cytopathologist would examine all 22 FOVs using a robotic microscope. If any of the fields were considered to be abnormal, then the entire slide was screened under a conventional microscope [68,69]. However, the slide could be classified as negative if all the FOVs were deemed normal. Next, another automated system was the FocalPoint GS imaging system, primarily known as AutoPap, which was trained on an image analysis algorithm to examine the aggregated risk of abnormality in whole slides. The screening system used a 25% cutoff value, which means that abnormal cell counts less than 25% were classified as negative without validated by the cytopathologists [68,69]. In 2001, it was approved by the FDA for the use of SurePath liquid-based cytology. Later this system underwent some modification and was renamed as a guided screening (GS) system with a self-regulating microscope and was known as the FocalPoint GS Imaging System. This system displayed the 10 most likely abnormal FOVs along with one FOV containing glandular cells [68,69]. Any abnormality that appeared in the FOV during the cytopathological reviewing process required manual screening of the whole slide. Moreover, another screening test came onto the market to enhance the accuracy of testing without human involvement and provide an automated liquid-based Pap test preparation (BestPrep^®^) and digital slide imaging system (BestCyte ^®^ cell sorter), known as Best Cyte, from Cell Solutions (Greensboro, NC, USA) [68,69]. BestPrep incorporated positive sample spotting and barcoding. Staining and coverslipping were performed autonomously by the laboratory and the BestCyte cell sorter, as a very fast-moving scanner and imaging software system, was used to classify and exhibit digital images on a screen with high resolution [68,69]. The system would save the images of whole-cell settling areas and then select, classify, and display selected cells and cell clusters in galleries based on predetermined cytological classifications [68,69]. Moreover, one study showed that the BestCyte cell sorter imaging system and BestPrep liquid-based thin-layer Papanicolaou test performed similarly to ThinPrep with respect to manual assessment [68,69]. Over the last decade, researchers have been trying to apply machine learning and deep learning algorithms to make more accurate software that can increase diagnostic efficacy without human involvement; for instance, a Chinese researcher used Pap smear and liquid-based cytology-based datasets to train a CNN model that differentiated benign and malignant cervical cancer cells with an accuracy of 98.3% and an AUC of 0.99 without performance of a prior segmentation task [70].

#### 4.4.2. Application of AI in Non-GYN Cancer Cytology

**Figure 8:**
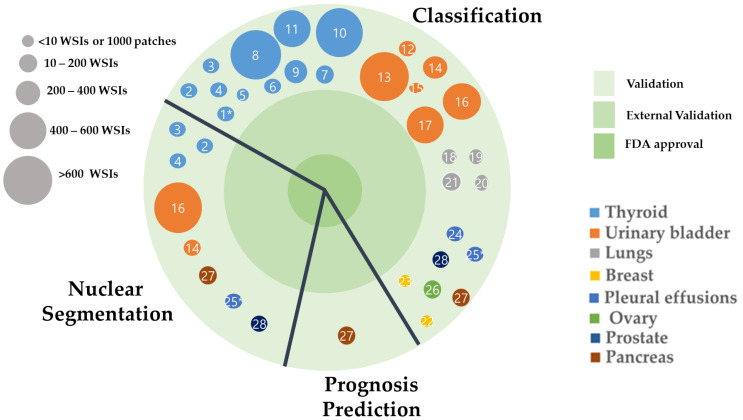
The application of AI models using cytological images applied for tumor classification, prognosis prediction, and nuclear segmentation. Published studies are denoted by serial numbers in Table 1. The studies are stratified according to the level of supporting evidence (outer circle, internally validated; middle circle, externally validated; inner circle, FDA-approved). * Indicates the dataset was in nuclei form instead of patches.

In the present study, we found that India is the country leading in the exploitation of cytology. Next, we found that thyroid cancer is being most intensely explored, followed by urinary bladder, lung, breast, pleural effusion, ovary, prostate, and pancreatic cancers. Most of the studies validated their models using internal validation. None of the models used external validation datasets or was approved by the FDA (Figure 8).

For thyroid cancer, only nine studies were included; most of the studies focused on the classification task, while a few studies focused on segmentation, with promising results. In our quantitative analysis, the Gopinath (3), and Guan et al. models showed higher sensitivity; Gopinath (1), Gopinath (2), and Gopinath (4) et al. models showed higher specificity; and the Savala et al. model showed higher accuracy. Gopinath et al. conducted a series of experiments for the classification of thyroid cancer [34,35,49]. In all four studies, the support vector machine (SVM) and similarly sized datasets (110 patch images) were used. In Gopinath (1), the Gabor filter bank was used for the segmentation task and then the SVM algorithm was used to predict malignancy of thyroid nodules with an accuracy of 96.7% and a specificity of 100% [34]. In Gopinath (2) et al., k-nearest neighbors (k-NN), an Elman neural network (ENN), and the support vector machine (SVM) were exploited. Next, discrete wavelet transform (DWT), Gray level co-occurrence matrix (GLCM), and Gabor filters were used to remove background noise during the segmentation task and then these images were separately classified by each algorithm. In contrast to other algorithms, the ENN classifier achieved the highest diagnostic accuracy [35]. In Gopinath (3), k-NN, decision tree (DT), ENN, and SVM classifiers were individually used to differentiate cancers. Then, multiple classifier fusion with majority voting rule and linear combination rules were exploited to increase the accuracy of the model, achieving an overall accuracy of 96.66%, which was higher than single classifiers [36]. In Gopinath (4), the researchers used an Elman neural network and an auto-associative neural network and achieved higher accuracy (96.66%) and specificity scores (100%) [49].

Moreover, in routine cytopathological diagnosis, it is very hard to differentiate FA and FC because of their similar microscopic structures. It is not feasible to differentiate only on the basis of cytological features; other parameters, such as extrathyroidal tumor extension, vascular invasion, capsule invasion, and lymph node metastases, etc., should also be considered [71]. Consistent with this, we found that the Savala et al. model was the best model because they trained the ANN model on various cytological features, such as cellularity, number of follicles, nucleoli, nuclear pleomorphism, nuclear margin, and morphometric analysis, which allowed the differentiation of FA and FC with 100% accuracy based only on cytological features [37]. However, a smaller dataset was a limitation [37]; future studies with larger datasets are required

There are many limitations to all the studies. Most of the studies did not disclose detailed information about pathologist involvement during the annotation process or about how cases of discrepancies during annotation were resolved, both of which affect the quality of datasets. Apart from these issues, most of the models were trained on cropped images rather than WSIs. It is always recommended to use WSIs because they consist of all biological information present in the cell, which can be missed out with the cropping of sections and models cannot be read exactly, leading to errors in diagnosis during clinical practice. In addition, external validation is always recommended to validate model efficacy, especially in the classification of cancer, and this was lacking in all of the studies.

**Figure 9:**
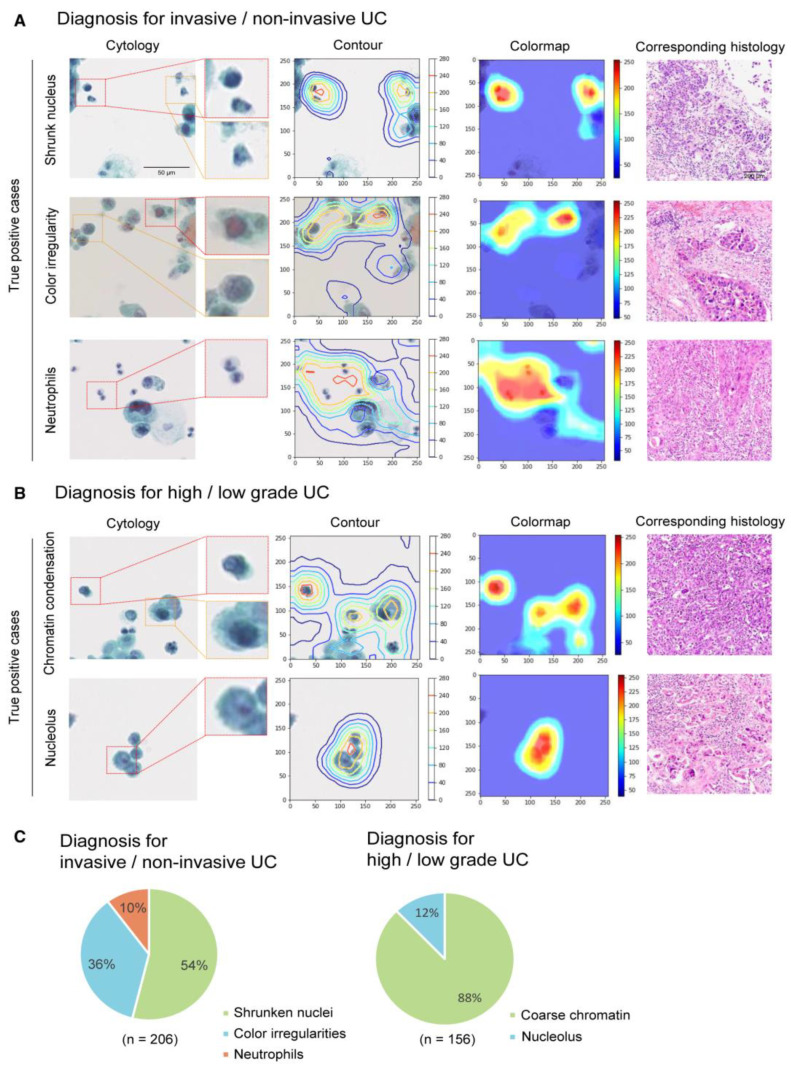
Example of stromal and nuclear grading using cytological images. Gradient-weighted class activation mapping (Grad-CAM) is illustrated. (**A**) Representative cytology images were observed using Grad-CAM and corresponding hematoxylin and eosin (H&E) histology images of invasive or noninvasive urothelial carcinoma (UC). Cytology images with a true-positive diagnosis contained cells with shrunken nuclei, nuclei with color irregularities, or neutrophil infiltration. (**B**) Representative cytology images were observed using Grad-CAM and corresponding H&E histology images of high-grade or low-grade UC. Cytology images with a true-positive diagnosis contained nuclei with coarse chromatin or an obvious nucleolus. (**C**) Pie charts indicate the proportions of findings associated with a true-positive diagnosis of stromal invasion or nuclear grading in the corresponding histology image. (Source: Figure 4 in Nojima et al. Reprinted with permission from the authors. Copyright (2021), Cancer Cytopathology, ACS journal [47].)

For urinary bladder cancer, there were only six studies included and a direct comparison of the models would be meaningless because of the differences in study design as well as in the datasets. In term of accuracy, only study by Murlidhharan et al. [42] showed higher accuracy (99.13%) for the classification. Another study by Nojima et al. presented a unique approach using the VGG model [47]. First, they classified malignant and benign cancer and then they classified stromal invasion and nuclear grading directly through the cytological images that corresponded to histological specimens with higher AUCs and F1 scores (Figure 9). Interestingly, their model classified malignant and benign cancer with a higher AUC as compared to existing models in this category [47]. However, in our opinion, a study from the USA was best because it used a larger dataset (2405 WSIs); the author developed a CNN-based model exploiting cell-level features as well as slide features to classify liquid-based urine cytology samples into five types, such as atypical, high-grade, low-grade, negative for high-grade, and suspicious for high-grade urothelial cancer, with an AUC of 0.88, sensitivity of 79.5%, and specificity of 84.5% [43].

**Figure 10:**
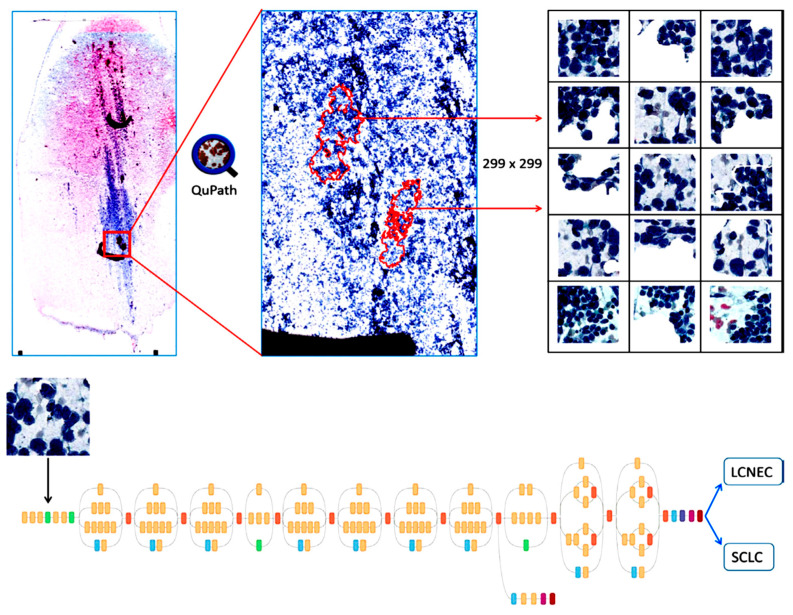
Example of cytological classification of lung cancer. (**Top** panel) Diagram showing the workflow for annotating and exporting image tiles using QuPath for subsequent input into the convolutional neural network. (**Bottom** panel) Schematic of the convolutional neural network architecture based on Google Inception V3 (adapted from https://github.com/tensorflow/models/tree/masterr/research/inception). (Source: Figure 2 in Gonzalez et al. Reprinted with permission from the authors. Copyright (2019), Cytopathology published by Wiley [28].)

For lung cancer, only four studies were applicable. Teramoto et al. performed a series of experiments with a different dataset. In 2017, the author constructed a CNN model for histological classification into three subtypes, SCLC, ADC, and SqC, with an accuracy of 71% [26]. Thereafter, in 2019, they extended their work for the classification of benign and malignant types with 79% accuracy [23]. In the next year, they found that by using a progressive growing generative adversarial network (PGGAN) the accuracy of the model was improved by 4% as compared to ImageNet software for classification in the same task [27], which was found to be the best model in terms of accuracy as per our analysis. Moreover, Gonzalez et al.’s was the best study in term of sensitivity and specificity according to our analysis; in this study, the author used Diff-Quik, Papanicolaou stains, and H&E images to train three algorithms that differentiated SCLC and LCNEC with higher sensitivity and specificity; however, the dataset used for the construction of the algorithm was very small (117 slides), which was not sufficient to conclude the algorithm’s robustness, such that further studies with a larger dataset and an external validation dataset are required [18]. The overview of their study design is shown in Figure 10.

Although the results for pleural effusion, ovary, prostate, and pancreatic cancer models were very promising for the classification task, the datasets were very limited, and the number of publications was very small. Therefore, it is impossible to compare model efficacy. Therefore, future studies with more standardized datasets are required.

### 4.5. Future Direction

Due to the digitization of tissue glass slides, the pathologist can differentiate the quantitative characterization of disease in precision medicine. In the histopathology field, the researcher’s focus has shifted from tumor classification to prognosis prediction and precision medicine based on direct use of histological images. For instance, Kather et al. constructed a deep learning model to predict microsatellite instability (MSI) using colon cancer histological images. They also found an association of MSIness with PDL-1 expression and interferon γ [47]. A similar kind of strategy has also been performed with respect to pancreatic cancer; atypical cases that were diagnosed as benign were associated with better survival than malignant cases (*p* = 0.46) [30]. A direct linking of cytological images with prognosis prediction outcomes remains largely unexplored in non-GYN. A future study using a similar kind of strategy is required.

### 4.6. Recent Advancements in Digital Slide Repositories

**Figure 11:**
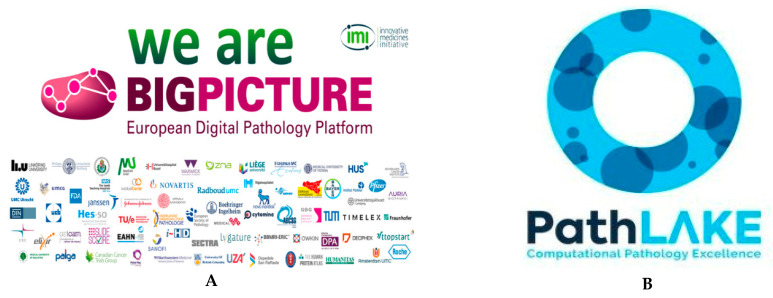

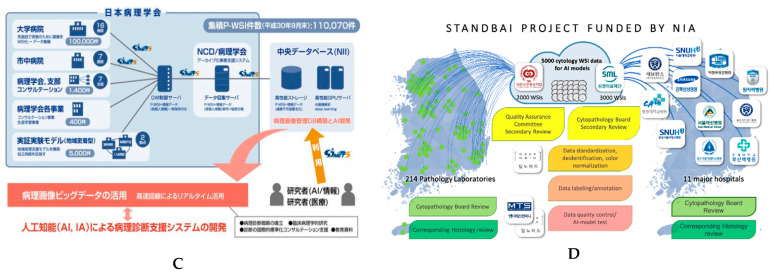
Representative figures of digital slides from repository projects: (**A**) BIG PICTURE, (**B**) Path LAKE, (**C**) JP-AID, and (**D**) NIA Data Dam.

To resolve the issue of the lack of publicly available datasets, many countries have taken a forward step. In Europe, the “BIGPICTURE” project has been launched this year (Figure 11A) and will continue for the next five years (with funding of EUR 70 M); the aim is to construct a library to store nearly 3 million digital images of human as well as laboratory animals and then use these data for the development of AI models for research purposes. The project involves various European hospitals, research centers, and major pharma industries. Similarly, in the United Kingdom, the “PathLAKE” project (with funding of EUR 50 M) involves the NHS, major industries, and university hospitals and will construct the world’s biggest library of annotated digital WSIs (Figure 11B). The main aim of this project is to develop robust AI models in the field of cellular pathology. In Japan, the “JP-AID” project launched in June 2018 aims to achieve four major objectives: (1) the establishment of AI medical image intelligence by merging image information from medical departments; (2) the standardization of pathological diagnosis reports; (3) the development of databases that can be used for diagnostic research; and (4) the establishment of a double-check system that exploits AI models (Figure 11C) In South Korea, the “NIA Data dam” project was launched this year with funding of USD 3.1 billion. It consists of 74 sub-projects and includes big industries, university hospitals, and computer labs. The STANDBAI sub-project (generation of STANdardized Digital pathology database of Biopsy and cytology for AI development) aims at the development of a cytological as well as a pathological dataset (Figure 11D). The purpose of this project is to establish public data for AI learning labelled by professionals and with verification to allow the possibility of developing an AI model for diagnostic assistance using constructed AI data.

Overall, the main objective of all these projects is to provide a dataset for the global researcher that can be used develop a robust artificial intelligence model that can diagnose disease precisely without human interference.

## 5. Conclusions

Overall, we have found that cytological examination is a crucial step in the initial diagnosis of cancer, although it shows limited diagnostic efficacy. AI models are still in the primary stage of development, even though the models show higher performance in terms of non-GYN cancer diagnosis using in-house data. Moreover, the lack of large, well-annotated datasets and Z-stacked images, along with the limited scale of datasets, were found to be major limitations in all the studies on the development of AI models examined. So far, none of the models has proved sufficient for the independent diagnosis of non-GYN cancer in the clinical setting. Future studies with large, multicenter datasets with high-quality annotations and external validation are required.

## Figures and Tables

**Table 1:** Characteristics of the AI models according to organ types using cytological image analysis.

No.	Organ	Author	Year	Country	Task	Staining and Preparation Method	Dataset	Pixel Level	Sampling	Z-Stacking Images	External Cross-Validation	Base Model	Performance	Pathologist Number
1	Thyroid	Varlatzidou [48]	2011	Greece	Classification Benign/Malignant	Pap	335 patients (32,887 nuclei)	1024 × 768	FNAC	ND	ND	ANN (LVQ)	Sens: 93.80% Spec: 94.11% Acc: 94.05%	NA
2		Gopinath (1) [34]	2013	India	Nuclear segmentation/ Classification Benign/Malignant	Pap	110 patches	256 × 256	FNAC	ND	ND	SVM/ k-NN,	Sens: 95% Spec: 100% Acc: 96.7%	ATLAS committee
3		Gopinath (2) [35]	2013	India	Nuclear segmentation/ Classification Benign/Malignant	Pap	110 patches	256 × 256	FNAC	ND	ND	SVM/ ENN/ k-NN	Sens: 90% Spec: 100% Acc: 93.3%	ATLAS committee
4		Gopinath (3) [36]	2015	India	Nuclear segmentation/ Classification Benign/Malignant	Pap	110 patches	256 × 256	FNAC	ND	ND	SVM/ ENN/ k-NN/ DT	Sens: 100% Spec: 90% Acc: 96.6%	ATLAS committee
5		Savala [37]	2017	India	Classification FA/FC	May Grunwald–Giemsa/H&E	57 cases (57patches)	NA	FNAC	ND	ND	ANN	Acc: 100% AUC: 1.00%	2
6		Gopinath (4) [49]	2018	India	Classification Benign/Malignant	Pap	110 patches	256 × 256	FNAC	ND	ND	ANN/ ENN	Sens: 95% Spec: 100% Acc: 96.7%	ATLAS committee
7		Sanyal [38]	2018	India	Classification PTC/non-PTC	Pap	370 patches	512 × 512	FNAC	ND	ND	CNN	Sens: 90.48% Spec: 83.33% Acc: 85.1%	NA
8		Dov [39]	2019	USA	Classification Benign/Malignant	Pap	908 WSIs (5461 patches)	150,000 × 100,000	FNAC	ND	ND	CNN (VGG-11)	Sens: 92% Spec: 90.5%	3
9		Guan [40]	2019	China	Classification Benign/PTC	LBC H&E	279 WSI (887 patch images)	224 × 224	FNAC	ND	ND	VGG-16/ Inception-V3	Sens 100% Spec 94.91% Acc: 97.6%	1
10		Range [22]	2020	USA	Classification Benign/Malignant	Pap	659 patients (908 WSIs) (4494 patches)	NA	FNAC	Yes	ND	Machine learning and CNNs	Sens: 92.0% Spec: 90.5% AUC: 0.93%	1
11		Frago-poulos [41]	2020	Greece	Classification Benign/Malignant	LBC Pap-stained	447 WSI (41,324 nuclei)	1024 × 768	FNAC	ND	ND	ANN (RBF)	Sens: 95.0%, Spec: 95.5%	NA
12	Urinary bladder	Murali-daran [42]	2015	India	Classification Benign/Low-grade/ High-grade	Pap	115 cases (115 patches)	NA	Urine sample	ND	ND	ANN	(1) All benign and malignant cases were diagnosed correctly (2) One of the low-grade cases was diagnosed as high-grade	2
13		Sanghvi [43]	2019	USA	Classification AU/HGUC/LGUN/SHGUC	NA	2405 WSIs (26 million cells)	150 × 150	Urine sample	Yes	ND	CNN	Sens: 79.5% Spec: 84.5% AUC: 0.88%	4
14		Vaickus [44]	2018	USA	Segmentation (Nucleus/Cytoplasm) Classification AU/BU/Sqc/Cry/Ery/Leu/BI/Deb	NA	217 WSIs (1.42 × 10^7^ million cells)	40,000 × 40,000	Urine sample	ND	ND	CNN (AlexNet/ResNet)	Acc: >95%	2
15		Zhang [46]	2020	China	Classification UC/SqC/DC/ IC/AU/SHGUC	Pap	49 cases 49 Images	NA	Urine sample	ND	ND	CNN	Identified abnormal urothelial cells	1
16		Awan [45]	2021	UK	Segmentation, Detection Classification Bc/IC/AU/SqC/SHGUC	LBC Pap	398 WSIs (9096 patches)	256 × 256 500 × 500 5000 × 5000	Urine sample	ND	ND	RetinaNet	AUC Atypical: 0.81 Malignant: 0.83	NA
17		Nojima [47]	2021	India	Classification Benign/Malignant Stromal invasion nuclear grading	LBC Pap	232 cases 466 WSIs (61,512 patches)	256 × 256 128 × 128	Urine sample	ND	ND	VGG16	AUC: 0.98, F1 score: 0.90 AUC: 0.86, F1 score: 0.82 AUC: 0.86, F1 score: 0.82	NA
18	Lungs	Teramoto (1) [26]	2017	Japan	Classification AdCC/SqCC/SCLC	Pap	76 cases (298 patches)	256 × 256	FNAC /Bronchoscopy	ND	ND	CNN	Acc: 71.1%	NA
19		Teramoto (2) [23]	2019	Japan	Classification Benign/Malignant	Pap	46 cases (621 patches)	224 × 224	FNAC /Bronchoscopy	ND	ND	CNN (VGG-16)	Sens: 89.3% Spec: 83.3% Acc: 79.2%	NA
20		Teramoto (3) [27]	2020	Japan	Classification Benign/Malignant	Pap	60 cases (793 patches)	256 × 256	FNAC /Bronchoscopy	ND	ND	CNN/ DCGAN/ PGGAN,	Sens: 85.4% Spec: 85.3% Acc: 85.3%%	NA
21		Gonzalez [28]	2020	USA	Classification SCLC/LCNEC	Diff-Quik/ Pap/H&E	40 cases (114 WSIs) (464,378 patches)	299 × 299	FNAC /Bronchoscopy	ND	ND	Inception V3	For Diff-Quik Model Sens: 1.00%, Spec: 87.5%, AUC: 1.00% For the Pap-stained Model Sens: 1.00%, Spec: 85.7%, AUC: 1.00% For the H&E model Sens: 1.00% Spec: 87.5% AUC: 87.5%	NA
22	Breast	Dey [24]	2011	India	Classification FAd/IDC/ILC	H&E	64 cases (64 patches)	NA	FNAC	ND	ND	ANN	ANN classified all the FA and ILC cases and six out of seven IDC cases	2
23		Subbaiah [25]	2013	India	Classification FAd/IDC	H&E	112 cases (112 patches)	NA	FNAC	ND	ND	ANN	Sens: 100% Spec: 100%	2
24	Pleural effusions	Barwad [31]	2011	India	Classification (Benign/Metastatic Carcinoma)	Giemsa/ Pap	114 cases (114 images)	NA	Pleural fluid	ND	ND	ANN	Acc: 100%	2
25		Tosun [32]	2015	USA	Nuclear segmentation/ Classification Benign/Malignant	Diff-Quik	34 cases (1080 nuclei)	NA	Pleural fluid	ND	ND	OTBL/ k-nearest	Acc: 100%	1
26	Ovary	Wu [29]	2018	China	Classification SC/MC/EC/CCC	H&E	85 WSIs (7392 patches)	227 × 227	FNAC	ND	ND	CNN (AlexNet)	Acc: 78.20%	2
27	Pancreas	Boroujeni [30]	2017	USA	Nuclear segmentation/ Classification/ Survival (Benign/Malignant/Atypical)	Pap	75 cases (277 images)	NA	FNAC	ND	ND	K-means clustering/MNN	Acc: 100% (Benign or malignant) Acc: 77% (Atypical cases classified as benign or malignant)	NA
28	Prostate	Nguyen [33]	2012	USA	Nuclear segmentation/ Classification Benign/Malignant	H&E	17 WSIs	Training 4000 × 7000 Testing 5000 × 23,000	NA	ND	ND	SVM/RBF kernel	Sens: 78%	NA

Abbreviations: FNAC: Fine-needle aspiration cytology, ND: Not done, ANNs: Artificial neural networks, LVQ: Learning vector quantizer, Sens: Sensitivity, Spec: Specificity, Acc: Accuracy, PTC: Papillary thyroid carcinoma, FA: Follicular adenoma, NA: Not available, FC: Follicular carcinoma, GAN: Generative adversarial network, SVM: Support vector machines, ENN: Elman neural network, k-NN: k-nearest neighbor, DT: Decision tree, LBC: Liquid-based cytology, RBF: Radial basis function, AU: Atypical urothelial cells, HGUC: High-grade urothelial carcinoma, LGUN: Low-grade urothelial neoplasm, SHGUC: Suspicious for high-grade urothelial carcinoma, BU: Benign urothelial cells, SqC: Squamous cells, Cry: Crystals, Ery: Erythrocytes, Leu: Leukocytes, BI: Blurry images, Deb: Debris, DC: Degenerated cells; UC: Urothelial cells, IC: Inflammatory cells, WSI: Whole-slide images, AUC: Area under the curve, H&E: Hematoxylin and eosin, OTBL: Optimal transport-based linear, CART: Classification and regression tree, SCLC: Small cell lung carcinoma, LCNEC: Large cell neuroendocrine carcinoma; AdC: Adenocarcinoma, SqCC: Squamous cell carcinoma, MNN: Multilayer perceptron neural network, FAd: Fibroadenomas, IDC; Infiltrating ductal carcinomas, ILC: Infiltrating lobular carcinoma, CNN: Convolutional neural network, SC: Serous carcinoma, MC: Mucinous carcinoma, EC: Endometrioid, CCC: Clear cell carcinoma.

## Data Availability

The data presented in this study are available on request from the corresponding author.
